# Drug-resistant profiles of extracellular vesicles predict therapeutic response in TNBC patients receiving neoadjuvant chemotherapy

**DOI:** 10.1186/s12885-024-11822-9

**Published:** 2024-02-07

**Authors:** Min Woo Kim, Hyojung Lee, Suji Lee, Sol Moon, Young Kim, Joon Ye Kim, Seung Il Kim, Jee Ye Kim

**Affiliations:** Department of Surgery, Yonsei University College of Medicine, 50-1 Yonsei-ro, Seodaemun-gu, 03722 Seoul, Republic of Korea

**Keywords:** TNBC, Neoadjuvant chemotherapy, Liquid biopsy, Predictive biomarker, Extracellular vesicles

## Abstract

**Background:**

Predicting tumor responses to neoadjuvant chemotherapy (NAC) is critical for evaluating prognosis and designing treatment strategies for patients with breast cancer; however, there are no reliable biomarkers that can effectively assess tumor responses. Therefore, we aimed to evaluate the clinical feasibility of using extracellular vesicles (EVs) to predict tumor response after NAC.

**Methods:**

Drug-resistant triple-negative breast cancer (TNBC) cell lines were successfully established, which developed specific morphologies and rapidly growing features. To detect resistance to chemotherapeutic drugs, EVs were isolated from cultured cells and plasma samples collected post-NAC from 36 patients with breast cancer.

**Results:**

Among the differentially expressed gene profiles between parental and drug-resistant cell lines, drug efflux transporters such as MDR1, MRP1, and BCRP were highly expressed in resistant cell lines. Drug efflux transporters have been identified not only in cell lines but also in EVs released from parental cells using immunoaffinity-based EV isolation. The expression of drug resistance markers in EVs was relatively high in patients with residual disease compared to those with a pathological complete response.

**Conclusions:**

The optimal combination of drug-resistant EV markers was significantly efficient in predicting resistance to NAC with 81.82% sensitivity and 92.86% specificity.

**Supplementary Information:**

The online version contains supplementary material available at 10.1186/s12885-024-11822-9.

## Background

Recent advancements in neoadjuvant chemotherapy (NAC) have broadened its applications beyond large aggressive tumors in breast cancer treatment. Candidates for preoperative systemic therapy are inoperable breast cancer, and operable breast cancer in selected patients who desire breast conservation with large primary tumor relative to breast size, who have HER2-positive disease and triple-negative breast cancer greater than clinical T2 or clinical N1. Current NAC strategies are highly personalized, considering the cancer subtype, stage, and molecular characteristics, to optimize treatment efficacy. Preferred regimens of NAC for HER2-negative breast cancer are dose-dense adriamycin and cytoxan followed by paclitaxel or weekly paclitaxel, and those for HER2-positive breast cancer are paclitaxel + trastuzumab, paclitaxel + carboplatin + trastuzumab (TCH), and TCH + pertuzumab. Moreover, these approaches might be integrated with both conventional therapies and immunotherapies to align with each patient’s unique cancer profile [1, 2]. The response to NAC remains a key prognostic indicator in several studies. Moreover, these developments in NAC protocols have notably increased the feasibility of breast-conserving surgeries, marking a significant shift in the breast cancer management landscape [3–5].

Clinically established therapeutics are highly effective in treating breast cancer; however, they present significant challenges. One major challenge is the limited therapeutic options for triple-negative breast cancer (TNBC), which is often resistant to chemotherapy and cross-resistant to other antitumor agents, suggesting multidrug resistance. In addition, the development of drug resistance to anthracycline + cyclophosphamide + taxane (ACT) combination chemotherapies undermines their therapeutic potential [6]. Moreover, some tumors relapse rapidly after NAC and surgery. Thus, a better understanding of the molecular mechanisms underlying drug resistance is urgently required to effectively treat TNBC.

Extracellular vesicles (EVs) are mediators of cell-to-cell communication, are surrounded by lipid bilayers, and are released from living cells. EVs carry real-time molecular information about their cell of origin, such as nucleic acids, proteins, and lipids. Among body fluid-derived EVs, tumor-derived EVs comprise vesicles released by highly heterogeneous breast cancer cells [7]. Therefore, the more accurately tumor-derived EVs can be isolated, the more accurately they can reflect the pathophysiological characteristics and behaviors of tumor cells [8]. An analysis of tumor-derived EVs collected from tumor tissues, especially at the early stages of drug resistance development, may offer insights into the possibility of screening and monitoring cancers, including breast cancer, and provide appropriate treatment options for patients in terms of precision medicine [9].

In this study, we developed several stable drug-resistant TNBC cell lines in vitro using a stepwise treatment strategy with chemotherapeutic agents for 28 weeks. We aimed to identify highly expressed biomarkers within EVs released from these cell lines and evaluate the clinical feasibility of EV-based assessments for predicting drug response in breast cancer patients receiving NAC.

## Methods

### Cell lines and anticancer drugs

The human TNBC cell lines HCC1395 (CRL-2324), MDA-MB-231 (HTB-26), and MDA-MB-468 (HTB-132) were purchased from the American Type Culture Collection (ATCC, Manassas, VA, USA). All cell lines were grown in RPMI-1640 (22400-089, Gibco, Carlsbad, CA, USA) supplemented with 10% heat-inactivated fetal bovine serum (FBS, 12483-020, Gibco), and 1% penicillin-streptomycin (15140-122, Gibco), and maintained in a humidified atmosphere of 5% CO_2_ in air at 37 °C.

Doxorubicin hydrochloride (Adriamycin, or Anthracycline chemotherapy drug, D4000) and docetaxel (Taxotere, or Taxane chemotherapy drug, D1000) were purchased from LC Laboratories (Woburn, MA, USA), and cyclophosphamide monohydrate (Cytoxan, NSC-26271) was purchased from Selleck Chemicals (Houston, TX, USA).

### Induction of chemotherapy resistance in breast cancer cells

Drug-resistant sublines of each TNBC cell line were derived from each original parental cell line by continuous exposure of low to high doses of anticancer drugs (1/120 IC_50_, 1/90 IC_50_, 1/60 IC_50_, 1/30 IC_50_, 1/10 IC_50_, and IC_50_) for over 6 months. Each parental cell line was treated with anthracycline + cyclophosphamide (AC, 1:10 molar ratio) for 72 h, repeated four times. Subsequent treatments with Taxotere (T) proceeded in the same manner, and this process was defined as one cycle. The cells were maintained for 6 months while the drug concentration was gradually increased and allowed to recover for an additional month.

### Evaluation of drug-resistant activity and growth rate

Sensitivity to chemotherapeutic drugs was measured with a colorimetric assay using MTT (M2003, Sigma-Aldrich, St. Louis, MO, USA). Following the treatment of cells with serial dilutions of AC or T for 72 h, MTT was added to each well and incubated for 4 h at 37 °C. Methanol was then added to each well and mixed for 30 min on an orbital shaker. The absorbance was recorded at 570 nm with a correction wavelength of 690 nm using a NanoDrop 3000 spectrophotometer (Thermo Fisher Scientific, Waltham, MA, USA). IC_50_ values were calculated using Prism v9.0.0 (GraphPad, San Diego, CA, USA).

To measure the growth rate of the derived cell lines, three groups of drug-resistant sublines and their parental cell line counterparts (1 × 10^5^) were seeded in 6-well plates and allowed to attach to the well surface. After attachment, the cells were counted every 24 h for 96 h using a cell counter (TC20, Bio-Rad, Hercules, CA, USA). The trypan blue exclusion assay was used to determine the number of viable cells in the cell suspension and evaluate the doubling time of the cells.

### RNA sequence analysis

The concentration and quality of total RNA were checked using a Qubit 2.0 fluorometer (Thermo Fisher Scientific). Total RNA (10 ng) was used to prepare strand-specific barcoded RNA libraries using the Ion AmpliSeq^™^ Transcriptome Human Gene Expression Kit (Thermo Fisher Scientific) following the manufacturer’s protocol. The Ion AmpliSeq Transcriptome Human Gene Expression Kit was designed for simultaneous targeted amplification of over 20,000 human genes using a single primer pool. A short amplicon (approximately 110 bp) was obtained from each target gene. AmpliSeq sequencing data were obtained using the Torrent Mapping Alignment Program optimized for Ion Torrent^™^ sequencing data to align raw sequencing reads against a custom reference sequence set containing all transcripts targeted by the AmpliSeq kit [10, 11].

### Transcriptome analysis

Differentially expressed genes (DEGs) were identified using DEGSeq (version 1.48.0) with p-values of < 0.05 and|fold changes|>2.5 threshold [12]. The function of each DEG was annotated based on the Biological Process Gene Ontology gene set (MSigDB collections, C5, BROAD Institute, Cambridge, MA, USA) and the Gene Reference into Function (GeneRIF, The National Center for Biotechnology Information) database. Volcano plots, bar plots, Venn diagrams, and heat maps were generated using ggplot2 (version 3.3.5) and the Complex Heatmap (version 2.10.0) software. All statistical analyses and visualizations were performed using R (version 4.1.2) and R Studio environment (release 077589bc).

### Flow cytometry for EV surface profiling

The surface profiles of cells and EVs were analyzed using flow cytometry (FACS LSR Fortessa system, Becton Dickinson, Franklin Lakes, NJ, USA) and specific antibodies against surface proteins. Cell samples were incubated for 30 min at 4 °C in the dark with one test dose of drug resistance detection antibodies (anti-MDR1, anti-MRP1, and anti-BCRP) and rinsed twice with FACS buffer to prevent excessive reactions; fluorescein isothiocyanate (FITC)-labeled secondary fluorescent antibodies were used to detect fluorescence signals.

Breast cancer-derived EVs bound to 3-µm microbeads (SPHERO^™^ Streptavidin Coated Particles, SVP 30 − 5, Spherotech Inc., Lake Forest, IL, USA) that were conjugated with biotinylated breast cancer-targeting antibodies (anti-EpCAM, anti-ITGA2, and anti-ITGAV), during incubation to enable the isolation of breast cancer-derived EVs, following protocols in a previous study [13]. After isolation from tumor tissue, the drug resistance detection antibody was then immobilized with a fluorescent detection antibody. Detailed schematics are presented in Additional File 1: Figure S1a and S1b.

### Confocal microscopy

To stain actin filaments, Alexa Fluor 594-conjugated phalloidin (A12381, Thermo Fisher Scientific) was used according to the manufacturer’s protocol, and DAPI (Vectashield, H-1200, Vector Laboratories, Inc., Newark, CA, USA) was used to stain cell nuclei. To confirm the presence of EVs, the captured EVs were detected using a primary PE-Cy7-labeled antibody against the general EV marker, CD63. EVs were also immobilized with a primary drug resistance detection antibody against MDR1 and a secondary FITC-labeled fluorescent antibody. Fluorescence images were obtained using a Zeiss LSM 700 confocal microscope (Carl Zeiss). The target and detection antibodies used in this study are listed in Additional File 1: Table S1.

### Transmission electron microscopy (TEM)

A drop of the EV sample was fixed in 2% glutaraldehyde-2% paraformaldehyde and placed on a Formvar carbon-coated grid for 15 s. Droplets were removed using filter paper, and a drop of 1% uranyl acetate was added for 15 s for negative staining, removed using filter paper, and washed with a drop of distilled water. The dried grids were observed using a transmission electron microscope (JEM-1011, JEOL, Tokyo, Japan) at an acceleration voltage of 80 kV equipped with a MegaView III CCD camera.

### Clinical characteristics of the participants

Clinical samples were obtained from subjects who visited Severance Hospital in South Korea in accordance with the guidelines of the independent Ethics Committee at the College of Medicine Yonsei University (IRB No. 4-2020-1292). Informed consent for the use of blood samples for research purposes was obtained from all patients. Pre-operative plasma samples were collected from the same patient before anesthesia. The criteria for subject eligibility in the analysis included (1) a confirmed pathological diagnosis of breast cancer, (2) collection of blood samples post-NAC and during the pre-operative period, and (3) hemolysis assessed before the isolation of EV to evaluate plasma sample quality. Details of the 36 individuals are shown in Additional File 1: Table S2.

### Receiver operating characteristic (ROC) analysis

ROC analysis of drug-resistant EV markers was performed on data from 20 patients with TNBC using MedCalc version 20.014 (MedCalc Software Ltd., Ostend, Belgium). We used univariate ROC analysis for each marker to obtain the area under the ROC curve (AUC) and evaluate the diagnostic power of drug-resistant EV marker combinations. Optimal criterion values were calculated by considering not only sensitivity and specificity, but also disease prevalence and costs of various decisions [14]. After performing a univariate ROC analysis on each combination of drug-resistant EV markers, we chose the “Best” combination with the highest AUC and the lowest standard error of AUC. Based on these calculations, we developed a combinatorial predictive score composed of MDR1, MRP1, and BCRP (combi-3) that was utilized to predict drug response in patients with breast cancer. Statistical analyses were performed using a one-way analysis of variance or Welch’s t-test.

## Results

### Establishment of TNBC cell lines with a drug-resistant phenotype

**Fig. 1:**
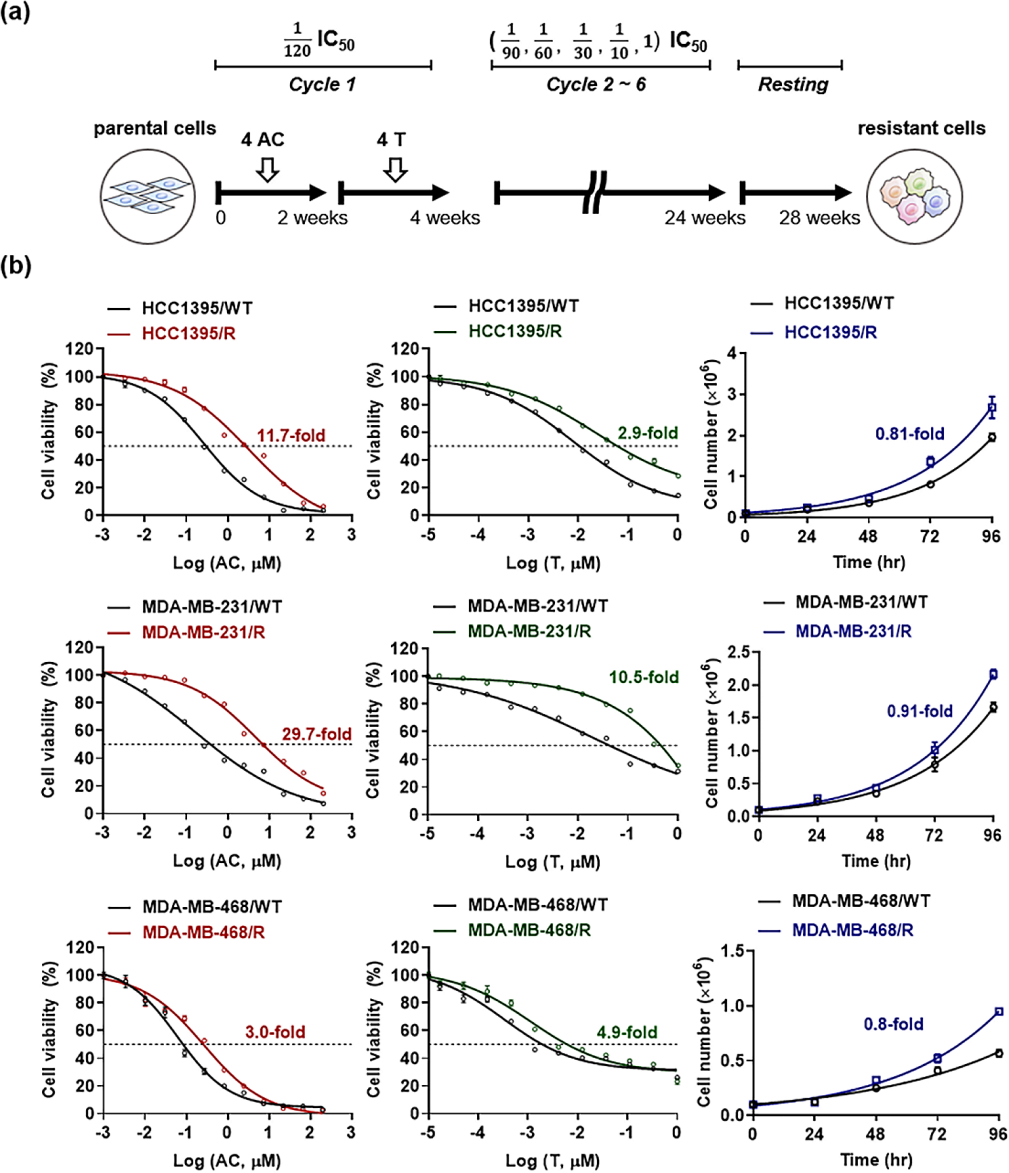
(**a**) Schematic illustration of the drug treatment process for the generation of drug-resistant cell lines. (**b**) Dose-response curves and IC_50_ values. Cell growth curves and their associated doubling times were measured. Data are expressed as Mean ± SEM (*n* = 5)

To generate chemotherapy-resistant variants of each TNBC cell line, the parental cell line was exposed to gradually increasing concentrations of chemotherapeutic drugs for 4 weeks per treatment cycle (Fig. 1a). Approximately 28 weeks and 56 passages were required for three cell lines (HCC1395, MDA-MB-231, and MDA-MB-468) to acquire stable drug resistance. The generation of drug resistance in TNBC cell lines was validated by morphological observations, dose-response analyses, and gene expression patterns at the transcriptional level. We generated dose-response curves and calculated IC_50_ values for both parental and drug-resistant (derived) sublines to determine whether the derived sublines acquired resistance to chemotherapy (Fig. 1b). As expected, the IC_50_ concentrations for drug-resistant sublines in response to AC or T treatment were significantly higher than those for the wild-type cell lines. Furthermore, doubling times were shorter in resistant cells than in wild-type cells, implying that the difference in proliferative capacity might be associated with the survival advantage of drug-resistant cells under chemotherapy. In summary, long-term exposure of TNBC cell lines to chemotherapeutic drugs was accompanied by distinct morphological changes, from 2.9- to 29.7-fold higher IC_50_ values and from 0.8- to 0.91-fold shorter doubling times in resistant TNBC sublines, thereby confirming the successful acquisition of drug-resistant phenotypes.

**Fig. 2:**
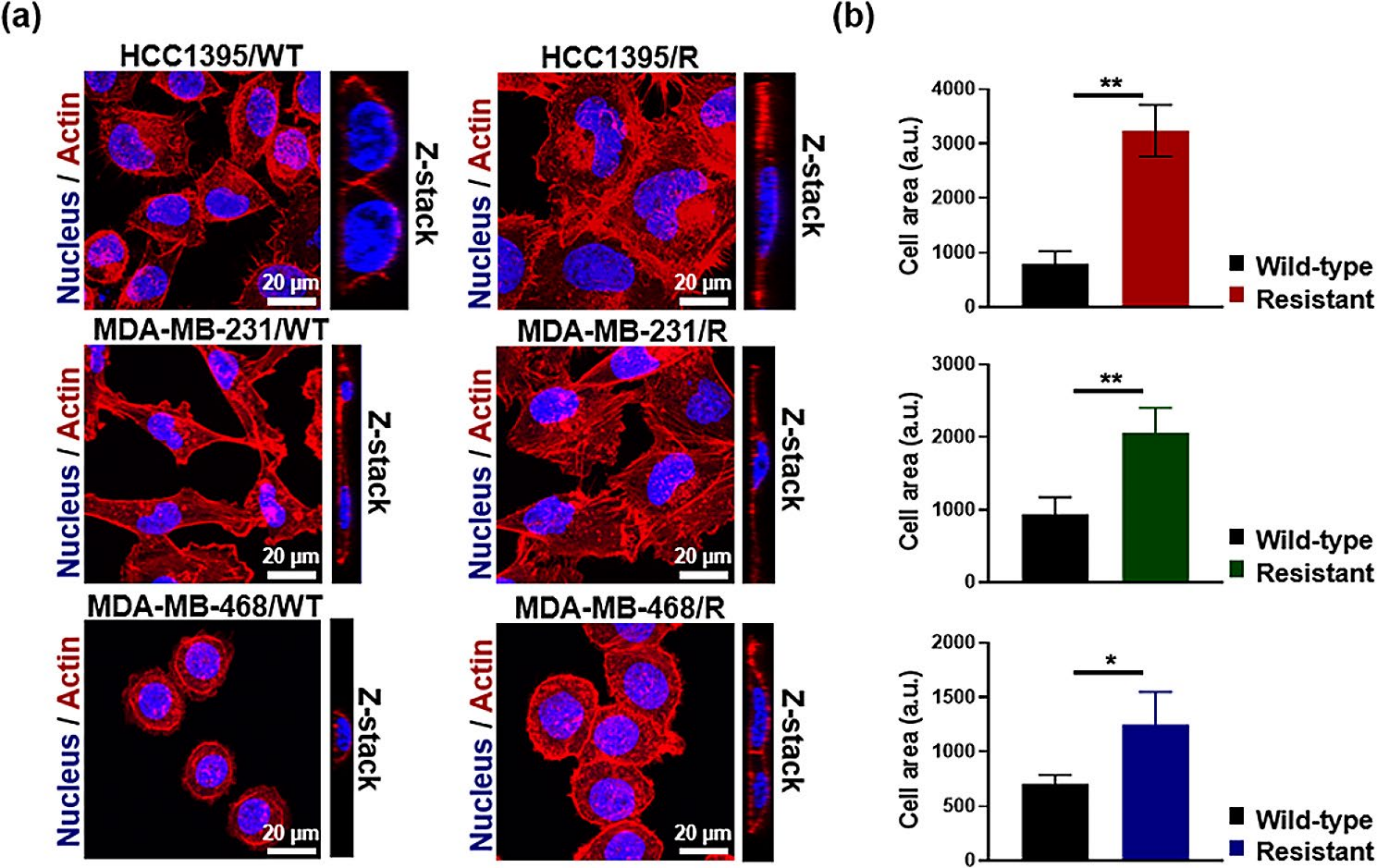
(**a**) Morphology of drug-resistant cell lines. Cell nuclei were stained with DAPI, and actin filaments were stained with phalloidin-Alexa Fluor 594. (**b**) Quantification of the area of TNBC cell lines. The cell areas were measured from three distinct cells in the image. Data are expressed as mean ± SD (*n* = 3). Significant differences between groups were determined using the Welch’s t-test (*p < 0.05 and **p < 0.01)

During drug treatment, we observed a gradual change in the shape of the TNBC cell lines. HCC1395, MDA-MB-231, and MDA-MB-468 cells spread more extensively in adherent monolayer cultures after acquiring resistance to anticancer drugs. The significance of the morphological changes was confirmed by the visualization of nuclei and actin filaments. We observed that the morphology of drug-resistant cells was distinct from that of parental cells of the same cellular age (Fig. 2a). Next, when we measured the area of TNBC cell lines, all resistant sublines exhibited enlarged nucleus/cytoplasm in monolayer proliferation compared to the parental cells (Fig. 2b).

### DEGs in resistant TNBC cell lines

**Fig. 3:**
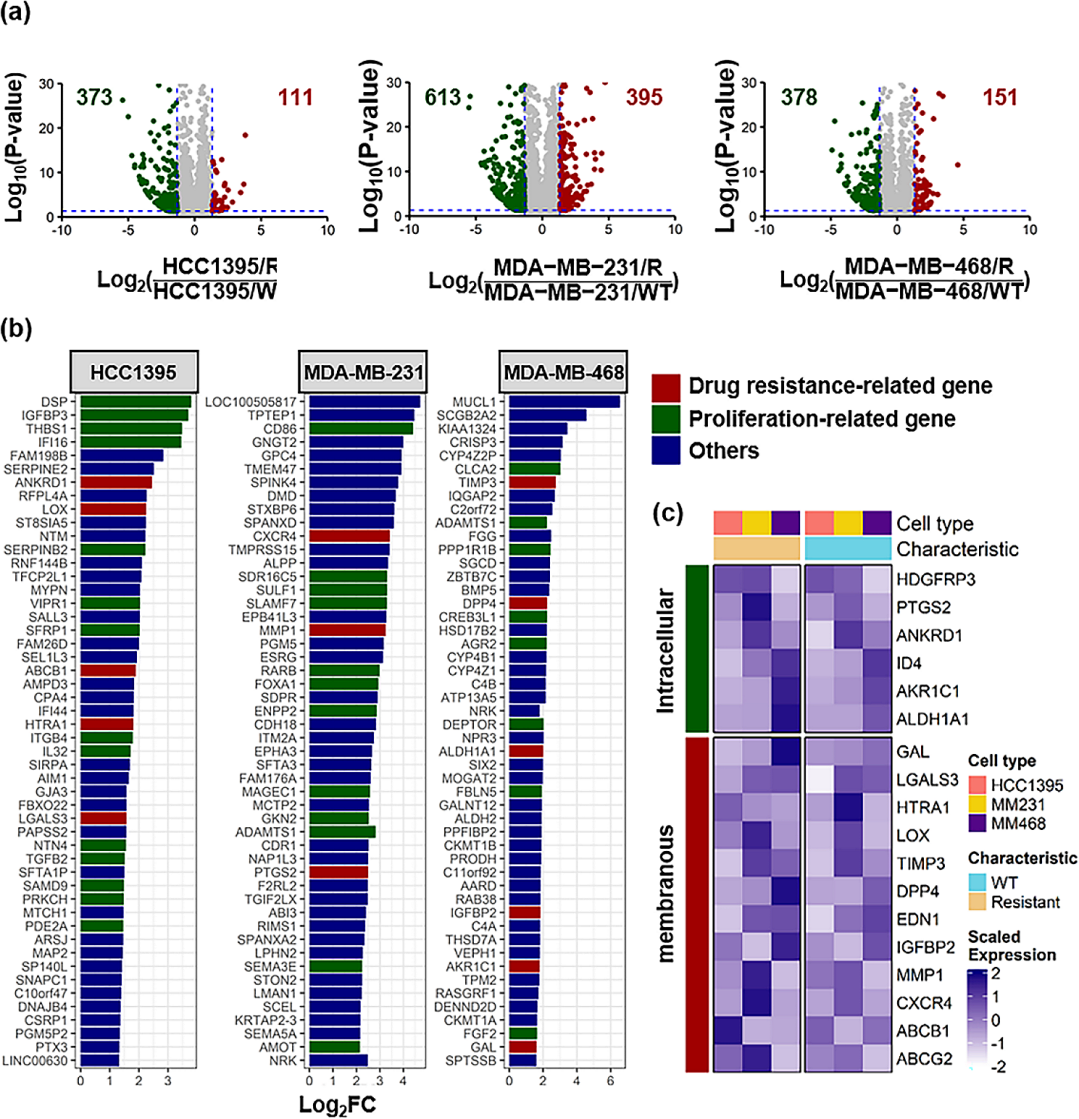
(**a**) Volcano plots [-log_10_(P-value) vs. log_2_(fold change)] for each comparison of wild-type and resistant HCC1395 (left), MDA-MB-231 (middle), and MDA-MB-468 (right) cells after long-term ACT treatment. The significant downregulated (green) and upregulated (red) transcripts are shown in each volcano plot. (**b**) A list of the 50 most significant genes in each upregulated DEG following long-term ACT treatment. The function of genes was annotated based on the GeneRIF database. (**c**) Heat map showing the relative abundance of selected drug resistance-related genes. The predicted locations of proteins encoded by drug resistance-related genes were categorized based on the Human Protein Atlas

To identify the differential abundance of transcripts in resistant TNBC, we performed RNA sequencing and transcriptomic analysis of the wild-type cell lines (hereafter referred to as HCC1395/WT, MDA-MB-231/WT, and MDA-MB-468/WT) and drug-resistant sublines (hereafter referred to as HCC1395/R, MDA-MB-231/R, and MDA-MB-468/R). A comparison of drug-resistant sublines with wild-type cell lines revealed a large number of significant differences in the volcano plot (Fig. 3a). Based on criteria [adjusted p-value ≤ 0.05, and fold change (FC) ≥ 2.5], 111, 395, and 151 upregulated DEGs, and 373, 613, and 378 downregulated DEGs were identified in HCC1395, MDA-MB-231, and MDA-MB-468, respectively. As the volcano plots indicate, in total, 657 significantly upregulated DEGs and 1,364 significantly downregulated DEGs were identified in the comparison between the wild-type and resistant groups; however, considering the limited expression level of downregulated DEGs not suitable for use as a diagnostic biomarker, they lost their significance in clinical analysis and were excluded from the candidate biomarker.

To further explore the biological activities of the top 50 upregulated DEGs, we cross-checked drug resistance- and proliferation-related genes found in GeneRIF, a functional annotation database. We identified 14 drug resistance-related DEGs and 34 proliferation-related DEGs, suggesting that these genes play important roles in the acquisition of chemotherapy resistance in TNBC (Fig. 3b). In addition to analysis using GeneRIF, Gene Ontology enrichment analysis for biological processes (GOBP) revealed that all resistant sublines had a high gene set variation analysis enrichment score in at least one GOBP term among drug transmembrane transport, drug transport, and cellular response to drugs. Notably, HCC1395 cells had positively enriched scores for all three GOBP terms, suggesting that these cells firmly acquired drug resistance, followed by MDA-MB-231 and MDA-MB-468 (Additional File 1: Figure S2). We also identified several gene sets potentially involved in drug resistance that contributed to the elevated enrichment scores in HCC1395 cells using gene set enrichment analysis and gene network analysis (Additional File 1: Figure S3). Importantly, many of the annotated genes found in these analyses encode membranous proteins, including drug efflux transporters (e.g., *ABCB1* and *ABCG2*), chemokine receptors (e.g., *CXCR4*), receptor tyrosine kinases (e.g., *EGFR*), and matrix metalloproteinases (e.g., *MMP1*), and so on (Fig. 3c). Among the membrane proteins that play vital roles in multidrug resistance, we focused on drug efflux transporters, the most frequently discovered molecules in chemoresistance [15].

### Induction of drug efflux transporters by chemotherapy agent

**Fig. 4:**
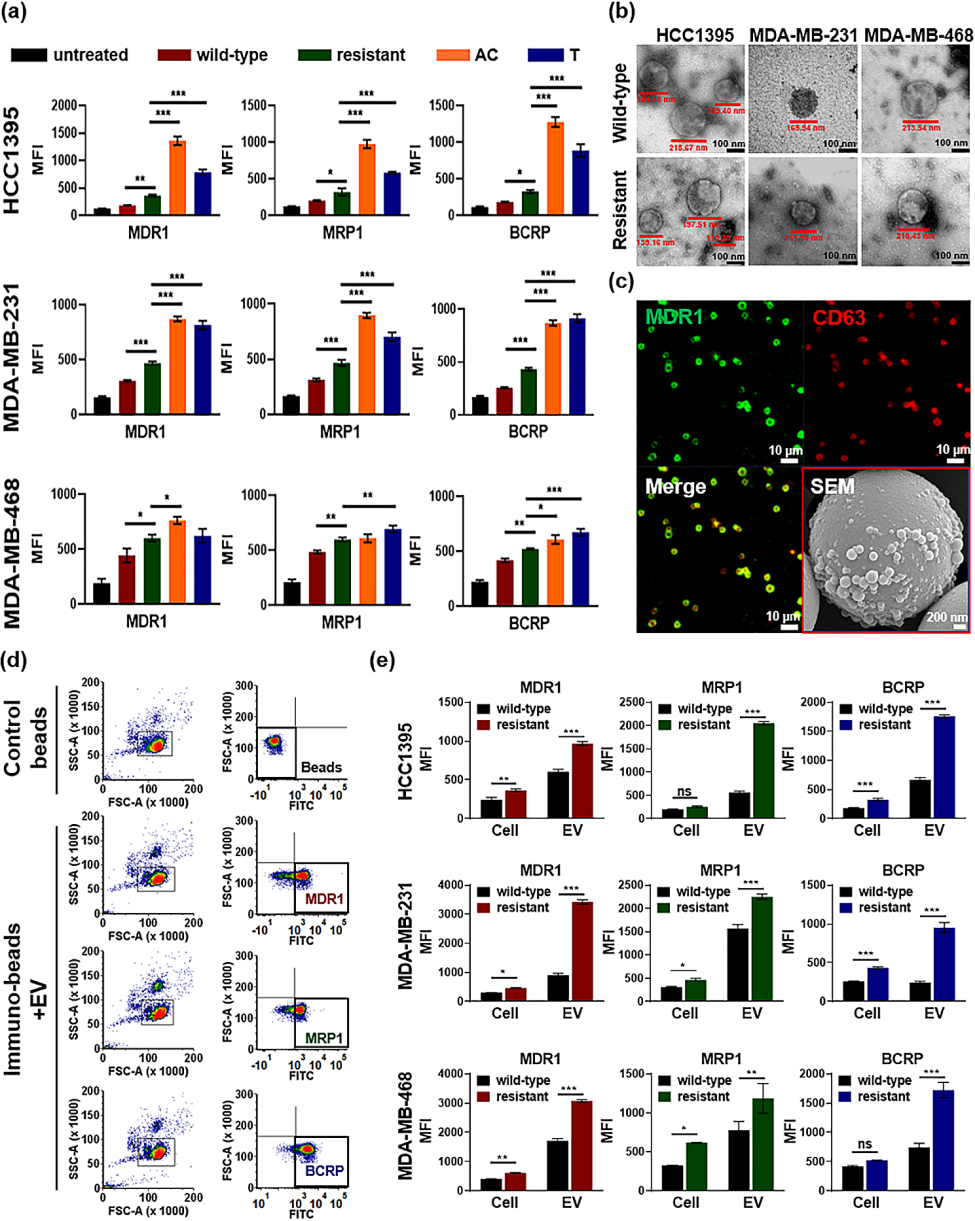
(**a**) Detection of drug-resistant proteins on the surface of HCC1395, MDA-MB-231, and MDA-MB-468 cells under different conditions. MFI for each target is shown on the y-axis: untreated control cells (black), wild-type cells (red), stable drug-resistant cells (green), drug-resistant cells after 48 h of AC treatment (orange), and drug-resistant cells after 48 h of T treatment (blue). Data are shown as mean ± SD of three independent experiments. Significant differences between groups were determined using one-way ANOVA (*p < 0.05; **p < 0.01; and ***p < 0.001). (**b**) TEM images of EVs extracted from each cell. Black scale bars represent 100 nm. (**c**) A representative confocal image of enriched EVs by immuno-beads with a red box highlighting a SEM image of EVs attached to immuno-beads. White scale bars represent 10 μm, except for the SEM image which represents 200 nm. (**d**) Flow cytometry gating strategies for drug-resistant EV markers (**d**) Comparison between wild-type and drug-resistant clones in cell lines and EVs. Significant differences between groups were determined using one-way ANOVA (ns, not significant; *p < 0.05; **p < 0.01; and ***p < 0.001)

Drug efflux transporters, also called ATP-binding cassette (ABC) transporter, are composed by 49 ABC genes arranged in eight human subfamilies [16]. Among those subfamilies, the three major types involved in cancer drug resistance are *ABCB1*, *ABCC1*, and *ABCG2*, which represent MDR1, MRP1, and BCRP proteins, respectively. Overexpression of these three ABC transporters can increase the efflux of drugs from cancer cells, thereby reducing the intracellular drug concentration [17]. We first investigated the expression levels of the three main drug efflux transporters in wild-type and resistant TNBC cells. Compared with those in the wild-type sublines, mean fluorescence intensity (MFI) values of MDR1, MRP1, and BCRP in the drug-resistant sublines were significantly higher (Fig. 4a, red vs. green column). Overall, stronger signals by drug efflux transporters were observed in the HCC1395, MDA-MB-231, and MDA-MB-468 drug-resistant sublines. Moreover, significant increases in drug efflux transporter expression were observed in the drug-resistant sublines after 48 h of AC or T treatment. Expression of these transporters was higher for HCC1395 and MDA-MB-231 cells and to a smaller extent for MDA-MB-468 cells in drug-resistant sublines. Long-term drug exposure in TNBC increased the immediate response capacity to drugs (Fig. 4a; green vs. orange or blue columns). These results indicate that transient and long-term exposure to chemotherapeutic agents induces the expression of cell surface drug transporters, which are primarily related to drug resistance.

### Detection of drug efflux transporters on EVs

Next, we hypothesized that EVs transfer their molecular cargo to adjacent cells which potentially influence the drug resistance profiles of tumor cells through the exchange of highly expressed drug efflux transporters. We designed an immunoaffinity-based breast cancer EV isolation method for cancer-derived EV surface profiling to evaluate whether experimentally accessible amounts of drug transporters could be detected in EVs. Before quantitative measurements, we verified that all EVs released from drug-resistant sublines had similar physicochemical characteristics. TEM and nanoparticle tracking analysis (NTA) confirmed the size, morphology, and concentration of EVs based on standard guidelines [18]. According to TEM observations, isolated EVs were approximately 100–200 nm in diameter and mostly spherical (Fig. 4b). When checking NTA results, the average particle sizes of EVs from drug-resistant sublines presented slightly larger particle sizes and higher EV concentrations than did EVs from wild-type cell lines (Additional File 1: Figure S4). Using confocal microscopy, we confirmed that a strong red fluorescent signal of the CD63 EV marker and a green fluorescent signal of the MDR1 drug transporter were detected on the EV attached to the microbeads, which indicates continuous activation at the surface of EVs (Fig. 4c). Scanning electron microscopy (SEM) was used to confirm the presence of tumor cell-derived EVs, along with their morphologies and sizes (Fig. 4c, red box).

Flow cytometric analyses identified three drug efflux transporters (MDR1, MRP1, and BCRP) in both the cell bodies and EVs released from parental cells. The fluorescence intensity of the control beads was used to set gating strategies for MDR1, MRP1, and BCRP. (Fig. 4d). Overall, the resistant clones were characterized by enhanced expression of drug efflux transporters. For MDR1, MRP1, and BCRP, the differences in MFI values between the wild-type and resistant clones significantly increased in EVs compared to the differences in the cell lines. The expression levels of drug efflux transporters in resistant EVs exhibited approximately 2.2 to 3.0-fold higher rates (Fig. 4e). These results suggest that the developed breast cancer-derived EV isolation method allows detection of membrane protein markers on the surface of EVs. Furthermore, EVs reflect the characteristics of the cells they are derived from and may be strongly relevant to inducing drug resistance after chemotherapy.

### Correlation analysis between drug-resistant EV markers with tumor response in TNBC patients treated with NAC

In this study, we evaluated the clinical utility of EVs as biomarkers for predicting pathological complete response (pCR) after NAC using drug-resistant EV marker selection and in vitro analyses. Among the 36 subjects who participated, pCR was defined as the absence of residual invasive cancer in the breast and lymph nodes 3–6 months after NAC, as assessed by pathological examination of tumor tissues post-surgery. The participants were divided into the pCR and non-pCR groups. Furthermore, breast cancer subtypes were determined via immunohistochemistry (IHC) of tumor biopsies following the guidelines established by Severance Hospital, which are based on the NCCN guidelines. Luminal A was defined as HR-positive, HER2-negative, with low Ki-67 levels (< 15%), whereas Luminal B was HR-positive, but with HER2-positive or higher Ki-67 levels (≥ 15%). The HER2 subtype was identified as HR-negative but HER2-positive, whereas the TNBC subtype was characterized as negative for both HR and HER2.

**Fig. 5:**
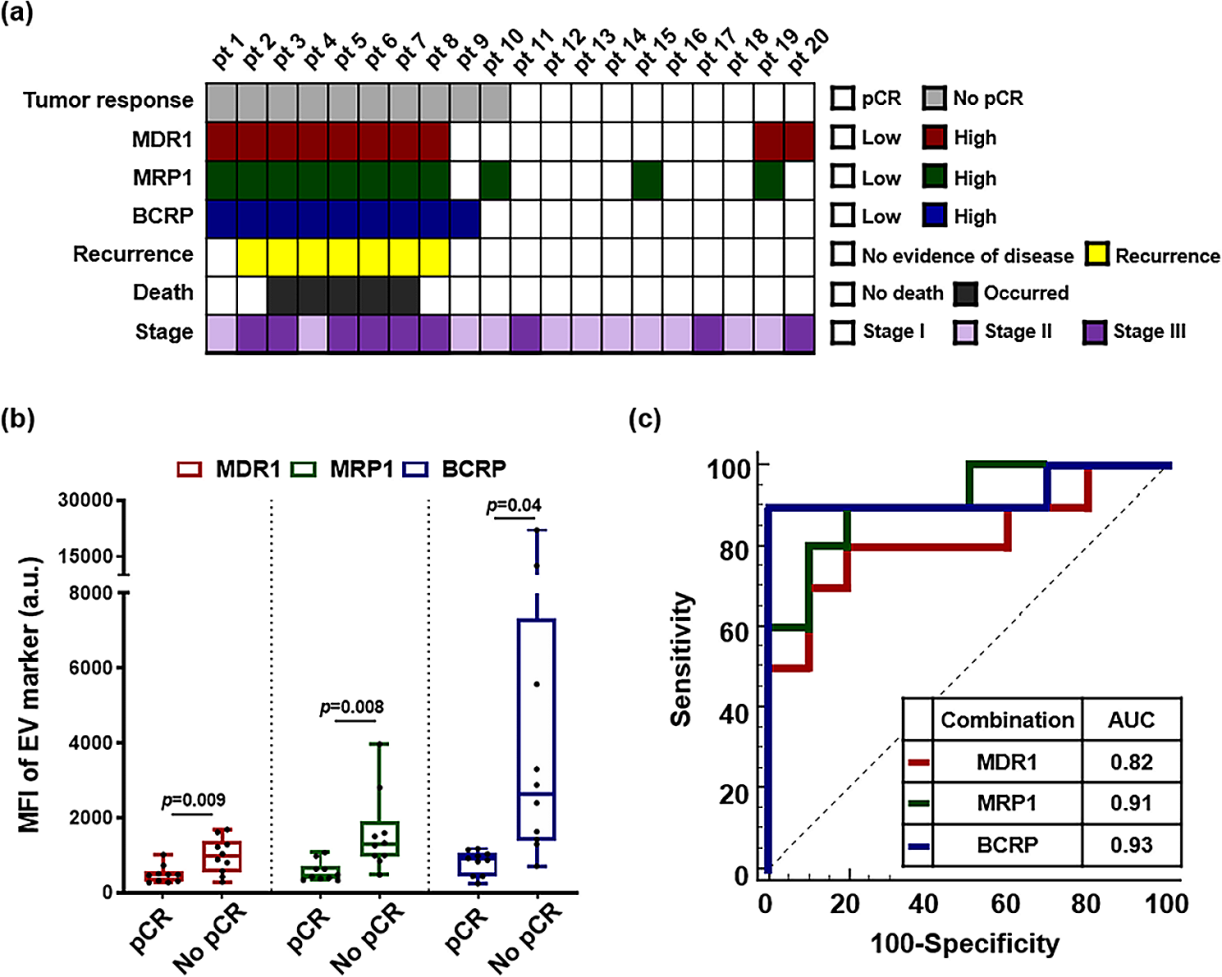
(**a**) Distribution of clinical variables and drug-resistant EV markers in patients with TNBC who underwent NAC. (**b**) Comparison of the MFI values for drug-resistant EV markers between patients with TNBC who responded to NAC and patients with TNBC who were resistant to NAC. Data are expressed as MFI in arbitrary units (a.u.) and represents the mean ± SEM of 20 individuals in each group. Statistical analysis was performed using the Welch’s t-test. (**c**) ROC curves for the three drug-resistant EV markers

We examined the expression levels of MDR1, MRP1, and BCRP on the surface of EVs from the plasma of 20 patients with TNBC who underwent NAC. Among the 20 patients, 10 showed residual tumors on surgical specimens and 10 achieved pCR after NAC. The expression of drug resistance markers in EVs in both groups was quantified objectively using the MFI values obtained by flow cytometry (Fig. 5a). The arbitrary cut-off values for the MFI of MDR1, MRP1, and BCRP were 506.8, 634.9, and 1175.8, respectively. At least one drug-resistant EV marker was highly expressed in all patients except two in the no pCR group, whereas most patients in the pCR group exhibited low expression levels of all drug-resistant EV markers (Additional File 1: Table S3). Notably, patients presenting with recurrence during follow-up or death showed elevated expression levels of all drug-resistant EV markers, implying a clinical correlation between drug-resistant EV markers and patient prognosis, as well as therapeutic response. The MFI values and distributions of all the EV markers differed significantly between the pCR and the no pCR groups (Fig. 5b). To assess the clinical utility of possible combinations of drug-resistant EV markers, we performed a ROC curve analysis on the same patient group and set the AUC cut-off for a good diagnostic value at > 0.8. The AUC values for MDR1, MRP1, and BCRP were 0.82 (*p* = 0.002), 0.91 (*p* < 0.001), and 0.93 (*p* < 0.001), respectively (Fig. 5c).

### Diagnostic potential of drug-resistant EV markers in all subtypes of patients with breast cancer treated with NAC

**Fig. 6:**
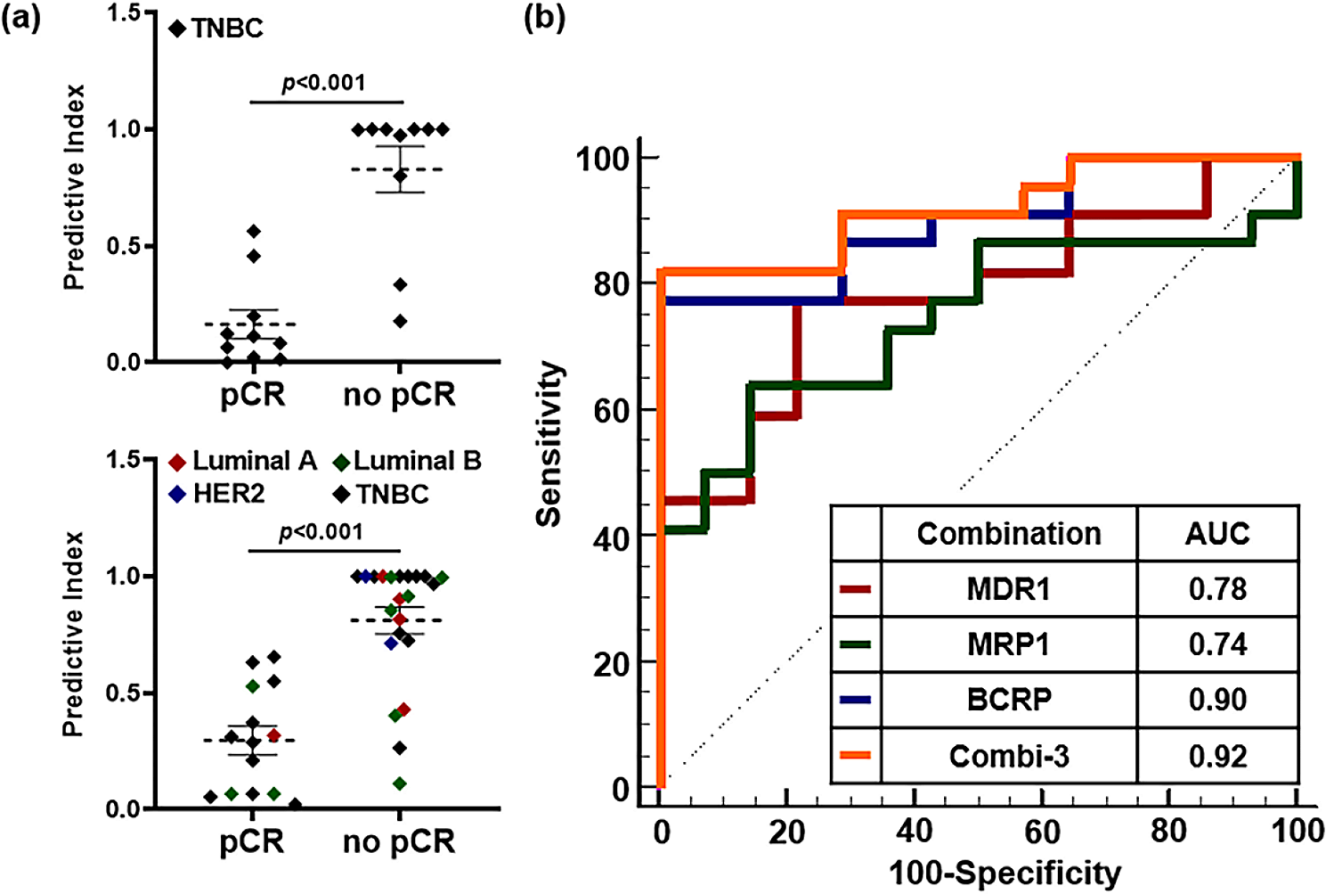
(**a**) Comparison of the combi-3 for drug-resistant EV markers between patients with breast cancer who responded to NAC and patients with breast cancer who were resistant to NAC. The top graph represents patients with TNBC (*n* = 20) and the lower graph represents patients with all subtypes (*n* = 36). Statistical analysis was performed using the Welch’s t-test. (**b**) ROC curves for the three drug-resistant EV markers and the combination marker

To better discriminate the therapeutic response and achieve the desired sensitivity and/or specificity of the markers, each biomarker was combined (Additional File 1: Figure S5). Using logistic regression, we identified the best combination of predictive biomarkers for drug response [19]. The patients with residual tumors after NAC had higher combi-3 expression, which was the optimal EV marker combination (*p* < 0.001; Fig. 6a, upper). Moreover, there were distinct differences in patients with breast cancer regardless of the subtype (*n* = 36), suggesting that the application of drug-resistant EV markers is not confined to TNBC but could be applied to all subtypes (Fig. 6a, lower). Next, we evaluated the performance of potential marker combinations in predicting drug responses based on the index using an optimal cut-off value of 0.631. The AUC values for MDR1, MRP1, and BCRP, when applied to all subtypes, were 0.78 (*p* = 0.002), 0.74 (*p* = 0.004), and 0.90 (*p* < 0.001), respectively. The AUC value of combi-3 was 0.92 (95% confidence interval, 0.830–1.000), with an 81.82% sensitivity and a 92.86% specificity (Fig. 6b). Therefore, we propose a more powerful model that combines EV markers to achieve the best diagnostic and prognostic accuracy for predicting treatment response in patients with breast cancer.

## Discussion

Within the past few decades, the standard method of evaluating the effectiveness of NAC has been to identify any residual tumor in surgical specimens; however, this is not possible before surgery. Approximately 10% of breast tumors do not respond to NAC, and resistant breast tumors, particularly TNBC, lead to disease progression and poor prognosis. Accordingly, to reduce disease progression, it is necessary to predict which patients will not respond to standard treatments [20]. Circulating EVs in the blood and intracellular communication vesicles with a lipid bilayer ranging in size from 50 to 300 nm are essential for tumorigenesis, development, progression, and metastasis [21]. Multiple technologies have been developed for tumor-derived EV detection and analysis (e.g., immunoaffinity-based capture) and have greatly advanced our understanding of tumor characteristics through liquid biopsy, despite the absence of tumor tissues. EVs can shuttle bioactive molecules such as proteins and a wide variety of genetic materials from one cell to another, leading to molecular transformations in recipient cells [22–25]. There may be an interplay or synergy between tumor cells in the acquisition of drug resistance via EV exchange. Therefore, we suggest a method that focuses on the role of EVs in multidrug resistance for early therapeutic response prediction and therapy monitoring.

Our study revealed the dynamics of the epigenetic changes that lead to drug resistance after chemotherapy in a cell line model. Various molecular mechanisms are involved in chemoresistance; among these, we found significant changes in cancer stemness in drug-resistant TNBC models. For example, dysregulated TGF-β and Wnt signaling pathways may affect the overall progression to malignancy (Additional File 1: Figure S6). They are also known to play a positive role in promoting drug-resistant properties in the cancer stem cell (CSC) population [26, 27]. Furthermore, the cytotoxicity-induced morphological changes can be related to drug resistance. Cytoskeletal reconstruction induces biological changes in cancer cells with drug resistance [28]. After long-term treatment with chemotherapeutic drugs, actin stress fibers change, with a distinct feature showing the migratory dynamics of cell spreading [29]. All these phenotypic features can be predominantly attributed to their tumorigenic potential and multidrug resistance. Moreover, we focused mainly on the overexpression of ABC transporters, which results in drug resistance, a characteristic feature of CSC. Accumulating evidence from numerous studies, including ours, indicates that high expression of transmembrane proteins of this superfamily, such as MDR1, MRP1, and BCRP, is found in breast cancer, particularly in breast CSCs [30].

Despite the clear relevance of ABC transporters, which play a critical role in the development of multidrug resistance, clinical approaches for assessing these proteins in the development of drug resistance have not been successful and are yet to be elucidated [31, 32]. However, with the availability of the latest technology for EV analysis, we recommend re-evaluating the role of ABC transporters within EVs, not focusing on those in tumor tissues. Two notable singularities of our study are worth discussing. First, we found that a certain percentage of cells were stably resistant to NAC when a chemotherapeutic drug was administered continuously (Fig. 4a). A superficial explanation for this is that drug resistance may be induced by more than simple genetic alterations; dysregulation of major epigenetic factors in breast cancer may play a more important role. This hypothesis is consistent with several reports on the epigenetic control of CSCs and their influence on tumorigenesis, development, and responsiveness to therapy [33, 34]. Second, by using the enrichment of tumor-derived EVs, a higher expression of drug efflux transporters was observed in drug-resistant EVs than in wild-type EVs (Fig. 4e) [35–37]. This has been suggested earlier and may have clinical significance, as it indicates that resistant clones may induce bursts of ABC transport in EVs to enhance drug resistance.

Although a few studies have utilized miRNAs, EV concentrations, and cancer antigens to monitor and predict response to treatments [38–40], our study is a novel attempt to use tumor-derived EVs to assess ABC transporters isolated from the plasma of patients with breast cancer who are treated with NAC. In a retrospective study using plasma from 36 patients, considerable differences in the expression of MDR1, MRP1, and BCRP were detected between patients with pCR and those with residual tumors. The combination of these three parameters offered acceptable sensitivity, specificity, and accuracy in predicting the effectiveness of NAC through cumulative ROC analysis. Apart from the lack of understanding regarding the role of EVs in cancer stem cells and drug resistance, we suggest that this method is a better strategy for repeatedly screening molecular information to predict therapeutic responses. However, three minor limitations of this study merit further discussion. First, we performed NGS analysis for established drug-resistant cell models compared to the wild-type to identify drug resistance genes and validated their expression in EVs isolated from breast cancer patients undergoing NAC. This was due to our concerns regarding the challenges of accurately separating tumor-derived EVs and ensuring sufficient nucleic acid material for reliable NGS analysis. Further research is required to precisely separate tumor-derived EVs and analyze their contents. Second, there was a shortage of sufficient sample sizes to conduct significant cohort studies with high statistical validity. Another issue is the lack of longitudinal studies analyzing changes in EV markers over time in patients. Future studies will advance to larger patient groups and address how EVs crosstalk with other tumor cells of different phenotypes to obtain drug-resistant characteristics, in addition to MDR1, MRP1, and BCRP.

## Conclusions

We established drug-resistant TNBC cell lines and investigated the genes involved in drug transport, such as MDR1, MRP1, and BCRP, which were highly expressed in resistant cell lines compared to that in their wild-type counterparts. The expression of MDR1, MRP1, and BCRP in breast cancer-derived EVs increased after in vitro chemotherapeutic treatment, particularly in drug-resistant cell lines. To investigate the clinical significance of drug-resistant EV markers in patients with TNBC receiving NAC, patients with residual tumors were found to have higher expression levels of MDR1, MRP1, and BCRP in EVs than in patients with a pathological complete response. Integrated analysis of MDR1, MRP1, and BCRP expression showed significant differences according to tumor response, not only in TNBC but also in other subtypes. In conclusion, our findings demonstrate that this EV marker combination could be a useful predictor for discriminating breast cancer patients with residual disease from those with no residual disease after NAC, especially in TNBC.

### Electronic supplementary material

Below is the link to the electronic supplementary material.

Supplementary Material 1

## Data Availability

The RNA-seq data generated here is available in the Gene Expression Omnibus database under the accession code GSE230273. All processed data generated in this study are included in the Supplementary Information.

## Abbreviations

AC: Anthracycline + Cyclophosphamide
ACT: Anthracycline + Cyclophosphamide + Taxane
ATCC: The American Type Culture Collection
AUC: Area Under The Curve
ABC: ATP-binding Cassette
BCRP: Breast Cancer Resistance Protein
CSC: Cancer Stem Cell
Combi-3: Combinatorial Predictive Score Composed of MDR1,MRP1,and BCRP
DEGs: Differentially Expressed Genes
EVs: Extracellular Vesicles
FBS: Fetal Bovine Serum
FC: Fold Change
GOBP: Gene Ontology Enrichment Analysis for Biological Processes
GeneRIF: Gene Reference into Function
IC50: Half-maximal Inhibitory Concentration
IHC: Immunohistochemistry
MFI: Mean Fluorescence Intensity
MRP1: MDR-associated Protein 1
MDR1: Multidrug Resistance Protein 1
NTA: Nanoparticle Tracking Analysis
NAC: Neoadjuvant Chemotherapy
pCR: Pathological Complete Response
ROC: Receiver Operating Characteristic
SEM: Scanning Electron Microscopy
T: Taxotere
TCH: Paclitaxel + Carboplatin + Trastuzumab
TEM: Transmission Electron Microscope
TNBC: Triple-negative Breast Cancer
