# Supplementary material for: Drug-resistant profiles of extracellular vesicles predict therapeutic response in TNBC patients receiving neoadjuvant chemotherapy
Source: BMC Cancer. 2024 Feb 7;24:185. doi: 10.1186/s12885-024-11822-9 (PMC10851537; doi:10.1186/s12885-024-11822-9)
Supplement: Supplementary file 1 — Supplementary Material 1 [file 12885_2024_11822_MOESM1_ESM.docx]

**Supplementary Material**

**Supplementary figures**


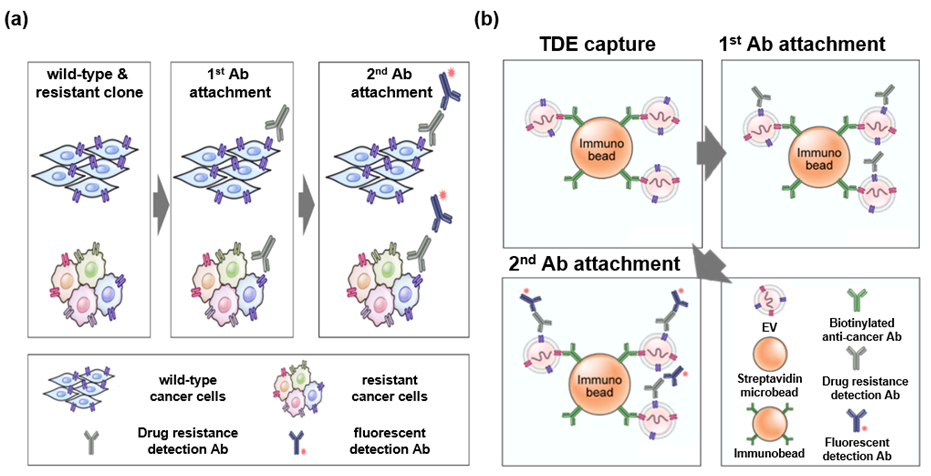


**Figure S1.** Detailed schematic of flow cytometry analysis. (a) Schematic of flow cytometry to measure the expression of three ABC efflux transporters (MDR1, MRP1, and BCRP) on the surface of cell lines. (b) Schematic of flow cytometry to measure the expression of three drug efflux transporters (MDR1, MRP1, and BCRP) on EVs using the immuno-affinity EV isolation method.


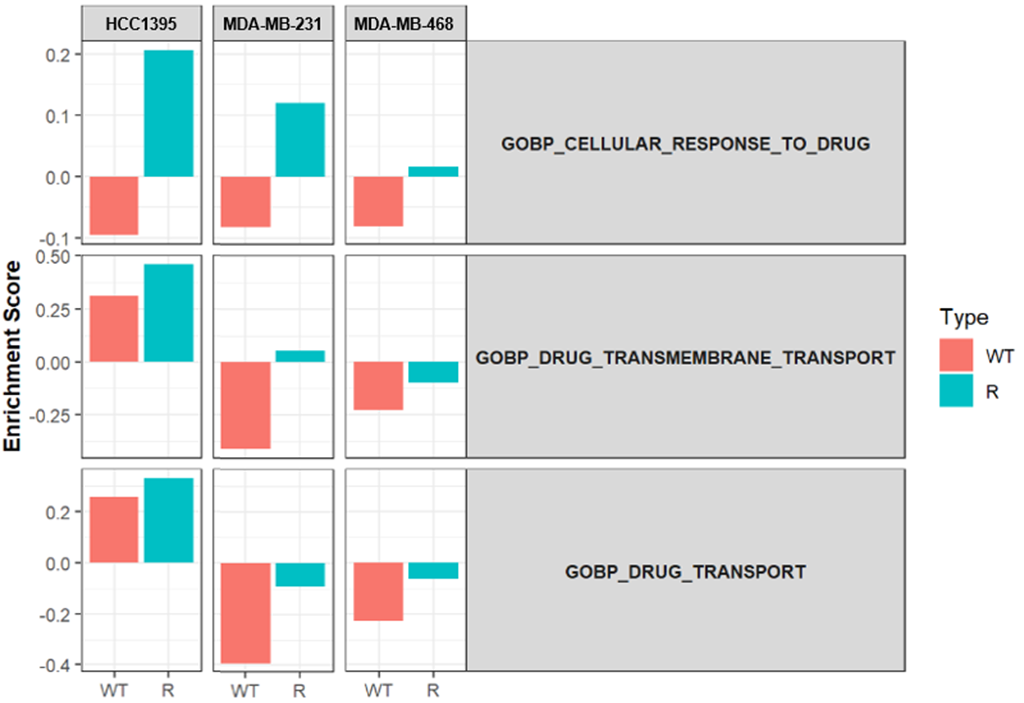


**Figure S2.** Bar plots of gene set variation analyses (GSVA) using three drug resistance-related Gene Ontology Biological Process (GOBP) terms including “cellular response to drug”, “drug transmembrane transport”, and “drug transport”.


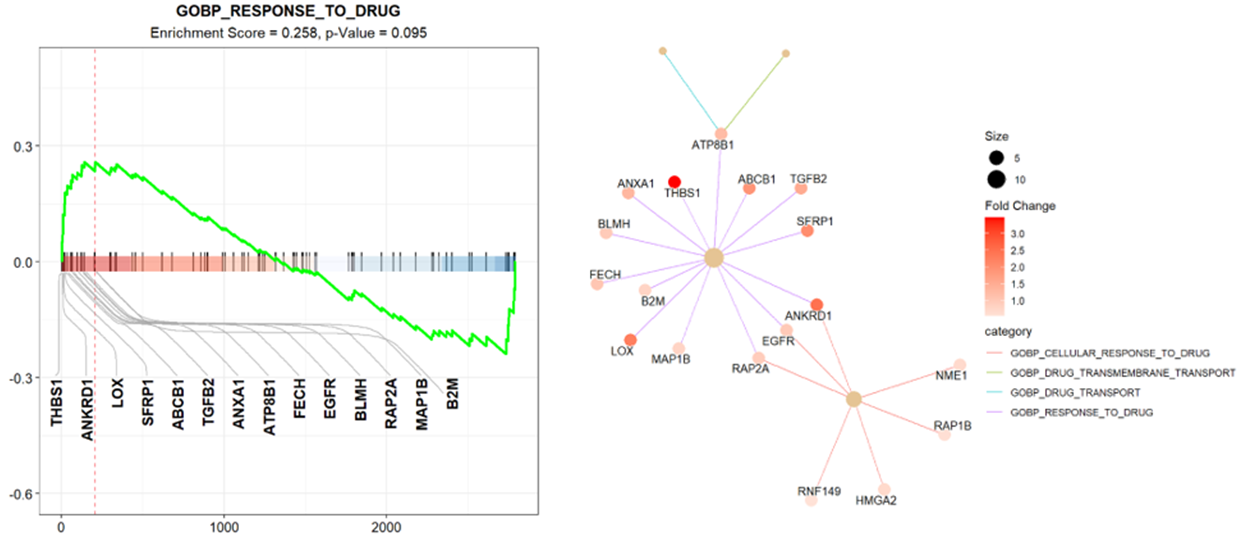


**Figure S3.** Gene set enrichment analysis (GSEA) enrichment score curves of response to drug and gene network analysis in the HCC1395/R cell line.


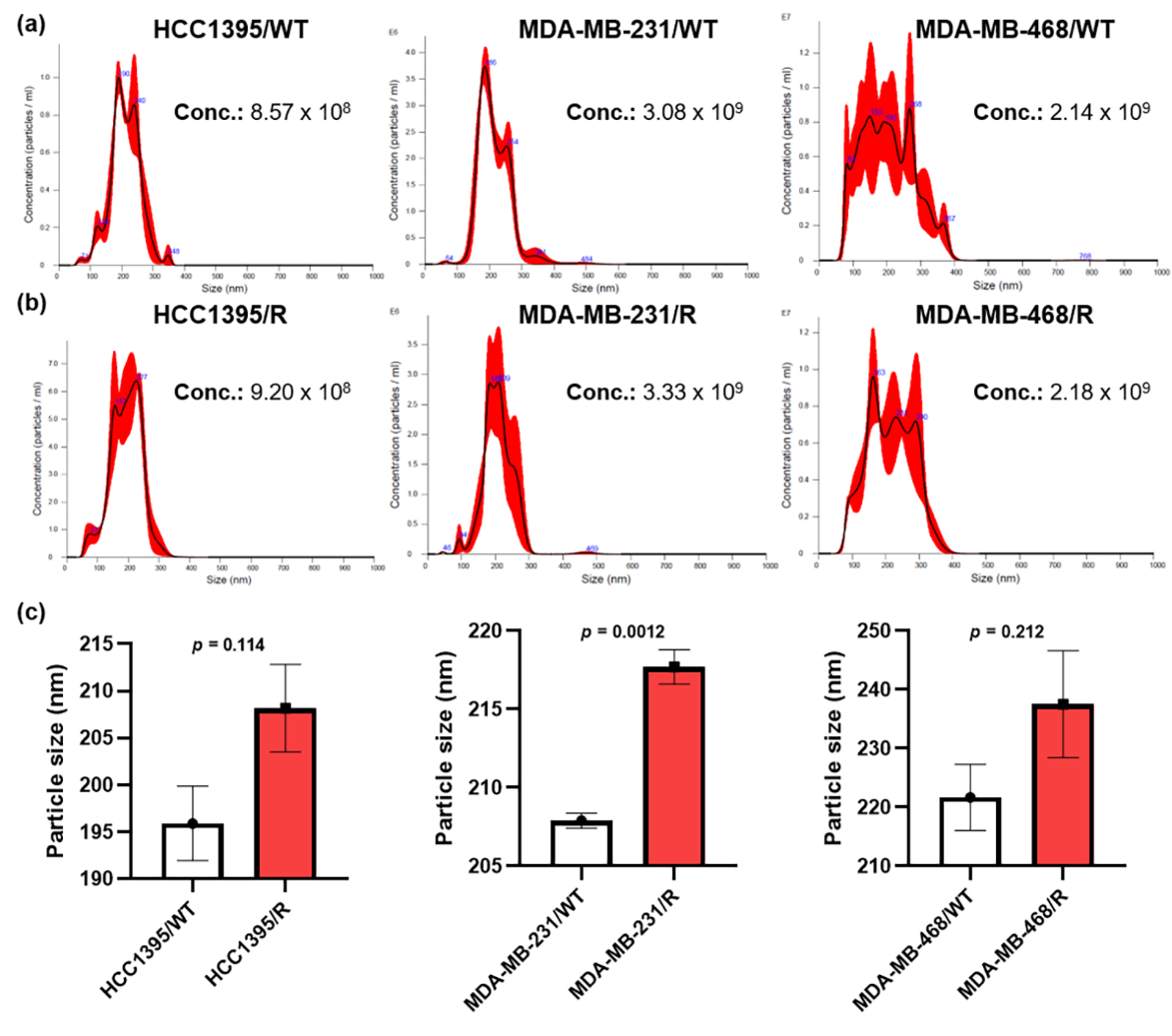


**Figure S4.** Nanoparticle tracking analysis (NTA) was performed on EVs released from both (a) wild-type (WT) cell lines and (b) drug-resistant (R) cell lines. All collected data were processed uniformly using identical parameters and representative images of size distributions were selected. Each sample underwent triplicate measurements across three separate experiments. (c) The average particle size for each group was compared and statistically analyzed utilizing the unpaired t-test (n=3).


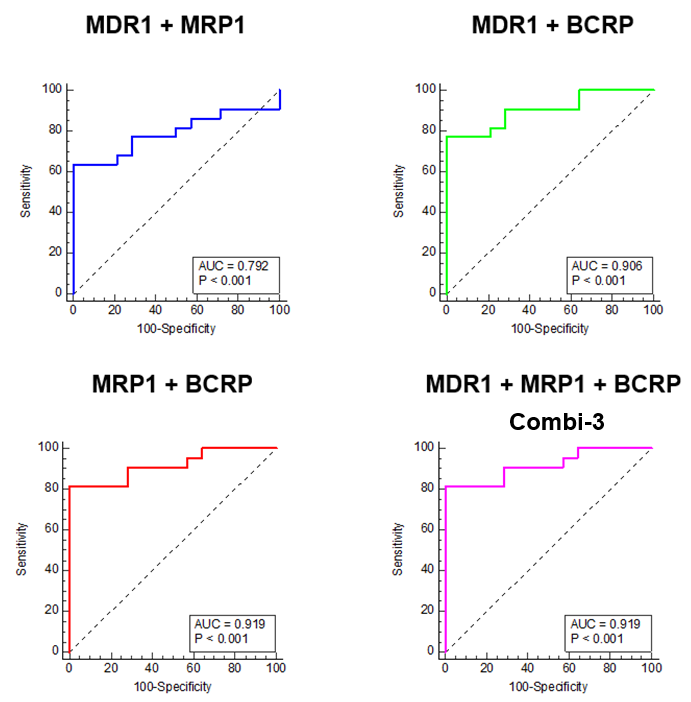


**Figure S5.** ROC curves for each drug-resistant EV marker or combination of three markers. The AUC represents drug resistance predictive performance.


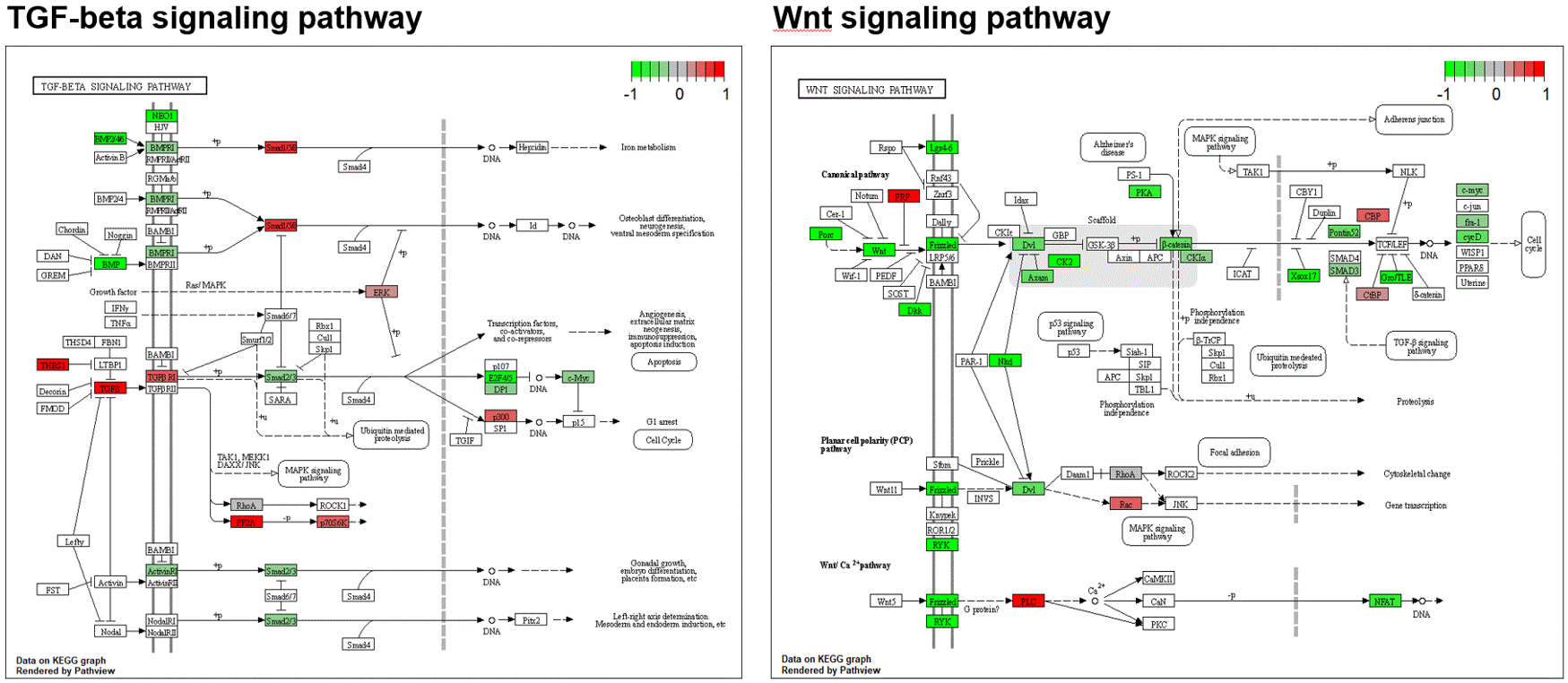


**Figure S6.** KEGG graphs of a metabolic pathway in the HCC1395/R cell line compared to those in the HCC1395/WT cell line.

**Supplementary tables**

**Table S1.** Antibody information

| **Target** | **Vendor** | **Catalog No.** | **Host species** |
| --- | --- | --- | --- |
| **Target antibody** | | | |
| Biotinylated integrin alpha 2 | R&D systems | BAM1233 | Mouse |
| Biotinylated integrin alpha V | STEMCELL | 60043BT | Mouse |
| Biotinylated EpCAM | Abcam | Ab79079 | Mouse |
| MDR1 (ABCB1) | Abcam | E1Y7S | Rabbit |
| MRP1 (ABCC1) | Abcam | D5C1X | Rabbit |
| BCRP (ABCG2) | Abcam | D5V2K | Rabbit |
| **Detection antibody** | | | |
| CD63-PE-Cy7 | BD Biosciences | 561982 | Mouse |
| FITC-conjugated anti-Rabbit IgG | Bethyl | A120-101F | Goat |

**Table S2.** Clinical information of study participants

| **Clinical variable** | **No pCR** | | **pCR** | | **Total** | | |
| --- | --- | --- | --- | --- | --- | --- | --- |
|  | **n** | **%** | **n** | **%** | **N** | | **%** |
| **All** | 22 | 61.1% | 14 | 38.9% | 36 | 100% | |
| **age (years)** |  |  |  |  |  |  | |
| ≤50 | 11 | 50.0% | 8 | 57.1% | 19 | 52.7% | |
| >50 | 11 | 50.0% | 6 | 42.9% | 17 | 47.3% | |
| **Clinical Stage** |  |  |  |  |  |  | |
| I | 2 | 9.1% | 1 | 7.1% | 3 | 8.3% | |
| II | 11 | 50.0% | 10 | 71.5% | 21 | 58.4% | |
| III | 9 | 40.9% | 3 | 21.4% | 12 | 33.3% | |
| **Subtype** |  |  |  |  |  |  | |
| Luminal A | 4 | 18.2% | 1 | 7.1% | 5 | 13.8% | |
| Luminal B | 6 | 27.3% | 3 | 21.5% | 9 | 25.0% | |
| HER2 | 2 | 9.1% | 0 | 0% | 2 | 5.6% | |
| TNBC | 10 | 45.4% | 10 | 72.4% | 20 | 55.6% | |
| **Distant recurrence** |  |  |  |  |  |  | |
| No | 15 | 68.2% | 14 | 100% | 29 | 80.6% | |
| Yes | 7 | 31.8% | 0 | 0% | 7 | 19.4% | |
| **Death** |  |  |  |  |  |  | |
| No | 17 | 77.3% | 14 | 100% | 31 | 86.1% | |
| Yes | 5 | 22.7% | 0 | 0% | 5 | 13.9% | |

**Table S3.** Mean fluorescence intensity of drug-resistant EVs from patients with breast cancer

|  | Class | | MFI | | | | Score | |  |
| --- | --- | --- | --- | --- | --- | --- | --- | --- | --- |
| Subtype | **Pt** | **0 : pCR / 1 : no pCR** | | **MDR1** | **MRP1** | **BCRP** | | **Combi-3** | |
| TNBC | 1 | 1 | | 886.52 | 994.56 | 22052.37 | | 1.00 | |
| TNBC | 2 | 1 | | 1286.12 | 1257.26 | 12557.8 | | 1.00 | |
| TNBC | 3 | 1 | | 1612.83 | 853.96 | 1426.72 | | 0.76 | |
| TNBC | 4 | 1 | | 1689.3 | 1328.25 | 3302.42 | | 1.00 | |
| TNBC | 5 | 1 | | 1042.07 | 2812.51 | 2877.45 | | 1.00 | |
| TNBC | 6 | 1 | | 804.5 | 1593.86 | 1636.01 | | 0.97 | |
| TNBC | 7 | 1 | | 582.43 | 1506.35 | 2398.14 | | 1.00 | |
| TNBC | 8 | 1 | | 1221.86 | 3972.91 | 1299.35 | | 0.72 | |
| TNBC | 9 | 1 | | 285.89 | 476.9 | 5569.45 | | 1.00 | |
| TNBC | 10 | 1 | | 422.65 | 984.91 | 706.71 | | 0.26 | |
| TNBC | 11 | 0 | | 1021.2 | 634.92 | 244.94 | | 0.02 | |
| TNBC | 12 | 0 | | 482.48 | 376.66 | 1175.86 | | 0.55 | |
| TNBC | 13 | 0 | | 461.42 | 630.43 | 435.31 | | 0.07 | |
| TNBC | 14 | 0 | | 322.61 | 391.35 | 977.43 | | 0.37 | |
| TNBC | 15 | 0 | | 484.51 | 330.76 | 830.32 | | 0.21 | |
| TNBC | 16 | 0 | | 307.49 | 1074.53 | 1033.02 | | 0.63 | |
| TNBC | 17 | 0 | | 506.87 | 479.19 | 917.21 | | 0.31 | |
| TNBC | 18 | 0 | | 269.13 | 427.67 | 869.57 | | 0.29 | |
| TNBC | 19 | 0 | | 272.87 | 337.98 | 435.06 | | 0.05 | |
| TNBC | 20 | 0 | | 732.62 | 979.64 | 1146.55 | | 0.65 | |
| Luminal A | 21 | 1 | | 645.33 | 1694.15 | 1304.28 | | 0.90 | |
| Luminal A | 22 | 1 | | 230.88 | 426.24 | 1010.84 | | 0.43 | |
| Luminal A | 23 | 1 | | 622.34 | 183.52 | 1555.48 | | 0.82 | |
| Luminal A | 24 | 1 | | 1729.75 | 832.5 | 3745.14 | | 1.00 | |
| Luminal B | 25 | 1 | | 277.5 | 209.42 | 656.38 | | 0.11 | |
| Luminal B | 26 | 1 | | 537.98 | 688.2 | 2352.09 | | 1.00 | |
| Luminal B | 27 | 1 | | 1123.94 | 2874.39 | 938.04 | | 0.85 | |
| Luminal B | 28 | 1 | | 3598.99 | 564.62 | 2849.74 | | 1.00 | |
| Luminal B | 29 | 1 | | 835.09 | 412.92 | 1729.38 | | 0.91 | |
| Luminal B | 30 | 1 | | 199.06 | 304.14 | 1014.54 | | 0.40 | |
| HER2 | 31 | 1 | | 1157.36 | 561.66 | 1390.09 | | 0.71 | |
| HER2 | 32 | 1 | | 1242.46 | 1158.1 | 14872.89 | | 1.00 | |
| Luminal A | 33 | 0 | | 265 | 292.2 | 940.17 | | 0.32 | |
| Luminal B | 34 | 0 | | 198.69 | 404.04 | 459.54 | | 0.07 | |
| Luminal B | 35 | 0 | | 198.69 | 404.04 | 459.54 | | 0.07 | |
| Luminal B | 36 | 0 | | 925 | 585.34 | 1162.91 | | 0.53 | |
